# A Simple Polypyrrole/Polyvinylidene Fluoride Membrane with Hydrophobic and Self-Floating Ability for Solar Water Evaporation

**DOI:** 10.3390/nano12050859

**Published:** 2022-03-03

**Authors:** Shenfeng Zhang, Jun Chen, Jixin Zheng, Xin Chen, Hongbo Xu, Florian Ion Tiberiu Petrescu, Liviu Marian Ungureanu, Ying Li, Gang Shi

**Affiliations:** 1 The Key Laboratory of Synthetic and Biological Colloids, Ministry of Education, School of Chemical and Material Engineering, Jiangnan University, Wuxi 214122, China; zsfjnu@126.com (S.Z.); jchenjnu@126.com (J.C.); 1052190311@stu.jiangnan.edu.cn (J.Z.); wxchengcheng@jiangnan.edu.cn (X.C.); liying@jiangnan.edu.cn (Y.L.); 2 MIIT Key Laboratory of Critical Materials Technology for New Energy Conversion and Storage, Harbin Institute of Technology, School of Chemistry and Chemical Engineering, Harbin 150001, China; 3 Department of Mechanisms and Robots Theory, Bucharest Polytechnic University, 060042 Bucharest, Romania; liviu.ungureanu@upb.ro

**Keywords:** photothermal membrane, intrinsic hydrophobicity, heat transfer, solar water evaporation, desalination

## Abstract

The traditional hydrophobic solarevaporator is generally obtained through the modification of alkyl or fluoroalkyl on the photothermal membrane. However, the modified groups can easily be oxidized in the long-term use process, resulting in the poor salt resistance and stability of photothermal membrane. In order to solve this problem, a simple polypyrrole/polyvinylidene fluoride membrane, consisting of an intrinsic hydrophobic support (polyvinylidene fluoride) and a photothermal material (polypyrrole), was fabricated by ultrasonically mixing and immersed precipitation. This photothermal membrane showed good self-floating ability in the process of water evaporation. In order to further improve the photothermal conversion efficiency, a micropyramid structure with antireflective ability was formed on the surface of membrane by template method. The micropyramids can enhance the absorption efficiency of incident light. The water evaporation rate reached 1.42 kg m^−2^ h^−1^ under 1 sun irradiation, and the photothermal conversion efficiency was 88.7%. The hydrophobic polyvinylidene fluoride ensures that NaCl cannot enter into membrane during the evaporation process of the brine, thus realizing the stability and salt resistance of polypyrrole/polyvinylidene fluoride in 3.5%wt and 10%wt NaCl solution.

## 1. Introduction

Solar vapor generation (SVG) is one of the most important ways to utilize solar energy [1,2,3,4,5]. In recent years, SVG has attracted much attention due to the increasing demand of fresh water [6,7,8,9,10,11,12]. The introduction of interface SVG, which is a self-floating solar absorber to float at the water–air interface, further improves the photothermal conversion efficiency of solar energy [13,14,15]. In the interface SVG system, the heat generated by solar energy can be confined to the evaporating surface, which helps minimize the heat loss of water reflux, thus improving the water evaporation rate and energy conversion efficiency of SVG system [16,17,18,19].

At present, in order to improve the photothermal conversion efficiency of the interface SVG system, researchers have carried out various advanced improvements. On the one hand, the photothermal conversion efficiency of evaporator can be improved by constructing a thermal insulating layer with water-transporting ability, which not only ensures that water can be quickly transferred to the surface of evaporator, but also avoids the loss caused by heat diffusion from the surface of evaporator to the water [20]. On the other hand, a balance between water transfer and evaporation can be achieved by regulating the surface wettability of the evaporator, so as to improve the photothermal conversion efficiency of the evaporator and avoid the convective thermal losses [21]. In addition, by regulating the hydrophilic functional groups of the evaporator, the equivalent evaporation enthalpy of water in the evaporator can be reduced, and then the photothermal conversion efficiency of the evaporator can be improved [22].

In the process of SVG, besides self-floating ability, hydrophobic membranes have better salt tolerance than traditional hydrophilic membranes [23,24]. The hydrophobic photothermal membrane has been applied to the interface SVG system, and its advantages are as follows. (1) Because the intermolecular force between hydrophobic materials and water is weak, the water vapor molecules are easy to separate from the hydrophobic membrane surface, which is conducive to accelerate the evaporation rate [25,26]. (2) Due to the self-floating ability of hydrophobic membrane, the bubbles will be generated at the contact interface between the membrane and water in the SVG process, which can reduce the loss of heat transfer to the bulk water and improve the photothermal conversion efficiency [27]. (3) By the reason of the hydrophobicity of photothermalmembrane, the salt water cannot pass through the membrane holes, and the water vapor can escape through the membrane holes. The salt grains are blocked at the interface of the hydrophobic layer, and then the salt grains are taken away by the reflux of water, thus effectively inhibiting the salt precipitation [28,29]. For example, Wang et al. assembled polypyrrole modified with fluoroalkyl silane on the stainless-steel mesh, to form a hydrophobic and self-healing photothermal membrane [30]. This hydrophobic membrane can spontaneously stay at the water–air interface and fully contact with water, so that solar radiation canprecisely heat the water at the gas–liquid interface. However, the hydrophobicityof the abovementioned photothermal membrane is mainly realized by surface modification with alkyl or fluoroalkyl. During the long-term use of photothermal membrane, it will be exposed to the environment of water and air, and under the action of strong ultraviolet light, the alkyl or fluoroalkyl will undergo oxidation reaction, resulting in the hydrophobic membrane into hydrophilic membrane [31,32,33].

Here, a novel polypyrrole/polyvinylidene fluoride photothermal membrane was designed by ultrasonically mixing and immersed precipitation. Then, the micropyramid structure was fabricated on the surface of membrane by soft imprinting [34]. Polypyrrole, as a photothermal conversion material, has wide waveband absorption. Polyvinylidene fluoride, as a hydrophobic support, has good self-floating and salt tolerance. Without unmodification, this composite membrane can maintain long-term stability in SVG process, due to the intrinsic hydrophobicity of polyvinylidene fluoride. The micropyramid structure has an antireflective ability, which can improve the utilization efficiency of incident light, and then improve the photothermal conversion efficiency of this system.

## 2. Materials and Methods

### 2.1. Materials

Polyvinylidene fluoride (PVDF), Dimethylformamide (DMF), Pyrrole, Ethanol, Ferric trichloride (FeCl_3_), Sodium hydroxide (NaOH) and Sodium chloride (NaCl) are all analytical reagents. All the materials were utilized as received without further purification.

### 2.2. Synthesis of PPy 

First, 1.622 g FeCl_3_ was added into 100 mL deionized water and stirred until completely dissolution. Then, 175 μL pyrrole was added into FeCl_3_ solution, and the reaction time was 3 h. After the reaction, the above solution was aged for 12 h, and polypyrrole (PPy) powder was obtained by filtration. The filtration was carried out by qualitative filter paper with a pore size of 30–50 μm. Finally, PPy powder was washed with anhydrous ethanol and deionized water for 3 times respectively, and dried in a vacuum oven at 60 °C for 8 h [35,36].

### 2.3. Fabrication of PVDF Membrane

First, 1 g PVDF powder was added into9 mL DMF and stirred until completely dissolved. Then, the above PVDF solution was poured into a hydrophobic mold. Finally, the PVDF membrane was obtained by quickly dipping the above mold into deionized water [37].

### 2.4. Fabrication of PPy/PVDF Membrane

First, 1 g PVDF powder was added into 9 mL DMF and stirred until complete dissolution. Second, 126 mg PPy powder was mixed into PVDF solution by the sufficient stirring. Then, the above solution was poured into a hydrophobic mold. Finally, the PPy/PVDF membrane was obtained by quickly dipping the above mold into deionized water.

### 2.5. Fabrication of PPy/PVDF Membrane with Micro-Pyramids

First, the Si template with micropyramid structure was obtained by etching Si wafer into alkaline solution for 30 min at 85 °C [38,39]. Next, 1 g PVDF powder was added into in 9 mL DMF and stirred until complete dissolution. Then, 126 mg PPy powder was mixed into PVDF solution by the sufficient stirring, subsequently, the above solution was poured into the hydrophobic Si template. Finally, the PPy/PVDF membrane with micropyramid structure was obtained by rapidly dipping the above Si template into deionized water.

### 2.6. Photothermal Performance Testing

First, a photothermal membrane with a diameter of 35 mm was floated on the deionized water in a container with an inner diameter of 39 mm. Then, under the irradiation of simulated 1 sun (1 kW m^−2^), the photothermal water evaporation experiment was carried out for 30 min. Meanwhile, the water evaporation mass change and the surface temperature of photothermal membranes were recorded at 5 min intervals. In addition, the photothermal water evaporation experiment of PPy/PVDF membrane in 3.5%wt and 10%wt NaCl solution was carried out. During the experiment, the temperature and humidity were kept at 26°C and 60% respectively.

### 2.7. Characterization

Scanning electron microscopy (SEM, S-4800, Hitachi, Tokyo, Japan) was used to observe the surface morphology of samples. Xenon lamp (CHF-XM-500W, PerfectLight Company, Beijing, China) was used to simulate the solar beam. Infrared camera (FLIR-E600), FLIRSystems Inc., Boston, MA, USA) was used to record the surface temperature of photothermal membranes. The reflectance, transmission, and absorption spectra of samples were measured by a UV-vis-NIR spectrophotometer (UV-3600plus, Shimazu Company, Kyoto, Japan) with a scanning wavelength range of 300 nm to 1100 nm. The valence state and electron transfer tests were performed using the Axis supra X-ray photoelectron spectrometer (XPS, Axis supra, Kratos, Manchester, UK).The molecular structures of the samples were characterized using a Fourier Transform Infrared Spectrometer (FTIR) (Nicolet 6700, Thermo Fisher Scientific, Waltham, MA, USA).The contact angle of samples was measured by Optical Contact Angle Measuring Instrument (OCA-40, Beijing Eastern-Dataphy Instrument Co., Ltd., Beijing, China).

## 3. Results and Discussion

### 3.1. Material Fabrication and Morphology

**Figure 1:**
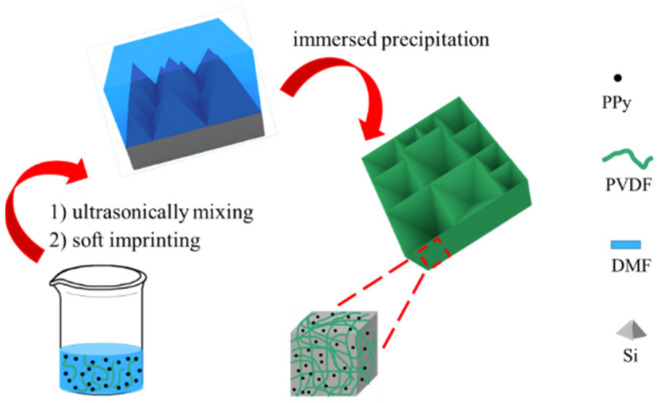
Schematic diagram of fabricating PPy/PVDF photothermal membrane.

**Figure 2:**
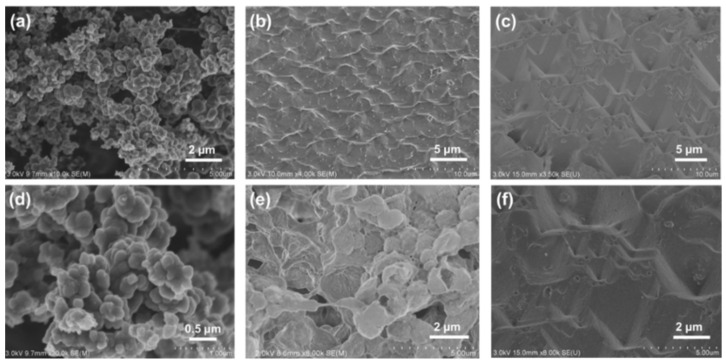
SEM images of (**a**,**d**) PPy powder; (**b**,**e**) PPy/PVDF membrane; (**c**,**f**) PPy/PVDF(P) membrane.

**Figure 3:**
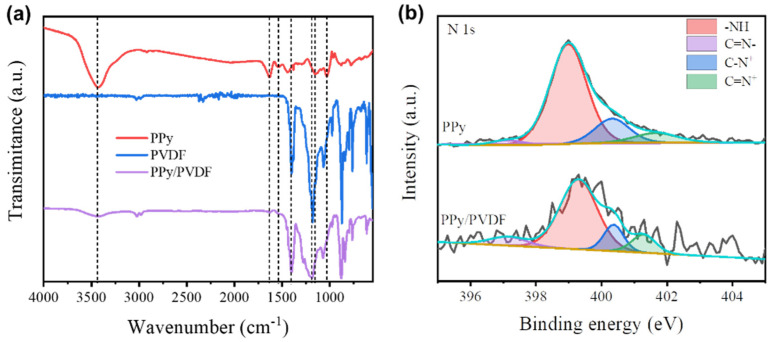
(**a**) FTIR spectraof PPy, PVDF and PPy/PVDF composite, and (**b**) XPS spectra of N 1s of PPy and PPy/PVDF composite.

Figure 1 shows the schematic diagram of fabricating PPy/PVDF photothermal membrane. First, PPy nanoparticles with a diameter of ~100 nm were synthesized by chemical oxidation polymerization, as shown in Figure 2a,d. Then, PPy nanoparticles were ultrasonically dispersed into PVDF solution, and poured into the mold. After standing, the above mold with PPy and PVDF was immediatelyimmersed into the deionized water to obtain PPy/PVDF membrane. The morphology of PPy/PVDF membrane is shown in Figure 2b,e. The rough structure with a fluctuation of ~1 μm was formed on the surface of PPy/PVDF, which is beneficial to increase the absorption efficiency of incident light. It can be seen from Figure 2e that the cross section of PPy/PVDF membrane is highly porous, which is favorable for the water vapor transmission. In addition, the porous PVDF networkplays a role in supporting and fixing PPy nanoparticles, which can ensure the long-term and stable photothermal conversion of PPy. Curves a, b, and c in Figure 3a are the IR spectra of PPy, PVDF, and PPy/PVDF, respectively. From the IR spectrum of PPy, a strong broad N–H stretching vibration absorption band at 3450 cm^−1^ is observed. The bands at 1631 cm^−1^ and 1544 cm^−1^ originated from C = C and C–C stretch peaks of ring-stretching modes. The bands at 1180 and 1050 cm^−1^ originated from C-N stretching vibration and C–H stretching vibration. The IR spectrum of PVDF shows that the bands at 1631 and 1544 cm^−1^ originated from CF and CF_2_ stretching vibrations. The IR spectrum of PPy/PVDF composite shows all of the above characteristic peaks, which proved that the PPy and PVDF composite were successfully prepared. Figure 3b shows the XPS spectra of PPy and PPy/PVDF. It can be seen that the deconvolution of N 1s peak of PPy leads to four main component peaks at 399.4, 400.83, 402.17 and 397.6 eV, which are attributed to N-H, C-N^+^, C = N^+^ and C = N, respectively. When PPy and PVDF is compounded, the binding energy of N-H bond shifts to 399.96 eV, indicating the strong hydrogen bond interaction between PPy and PVDF.

**Figure 4:**
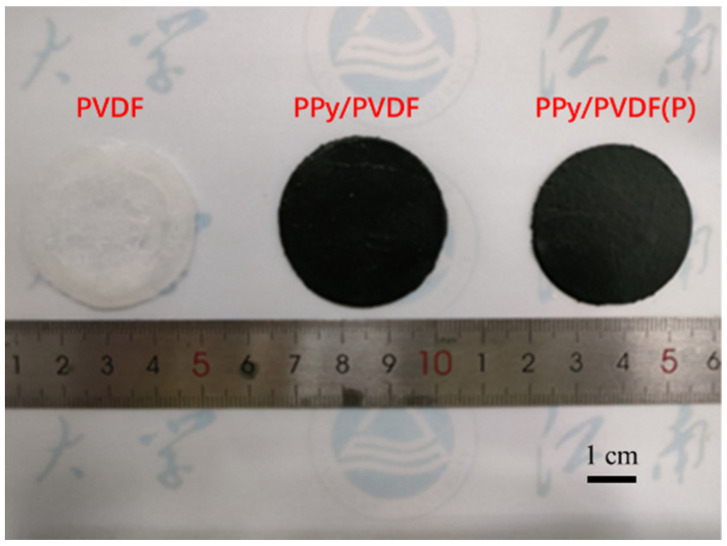
Photo of PVDF, PPy/PVDF, and PPy/PVDF(P) membranes.

In order to further enhance the photothermal conversion efficiency of PPy/PVDF membrane, a micropyramid structure with antireflection was fabricated on the surface of PPy/PVDF membrane by template method. The PPy/PVDF membrane with micro-pyramids is defined as PPy/PVDF(P). Figure 2c,f shows the average height of micropyramids is ~5 μm, which can effectively improve the absorption efficiency of incident light [40,41]. The photos of PVDF, PPy/PVDF, and PPy/PVDF(P) membranes are shown in Figure 4. Compared with the traditional SVG system, the advanced features of PPy/PVDF(P) are as follows. (1) PVDF in PPy/PVDF(P) has excellent salt resistance, which is conducive to its application in desalination. (2) The surface of PPy/PVDF(P) has an antireflective structure, which is conducive to improving its absorption efficiency of sunlight and thus improving its photothermal conversion efficiency. (3) Both PPy and PVDF are readily available commercial raw materials with low cost, which is conducive to the marketization of the system. (4) The fabrication process of PPy/PVDF(P) membrane is simple, which is conducive to its industrialization promotion.

### 3.2. Optical and Hydrophobic Property

**Figure 5:**
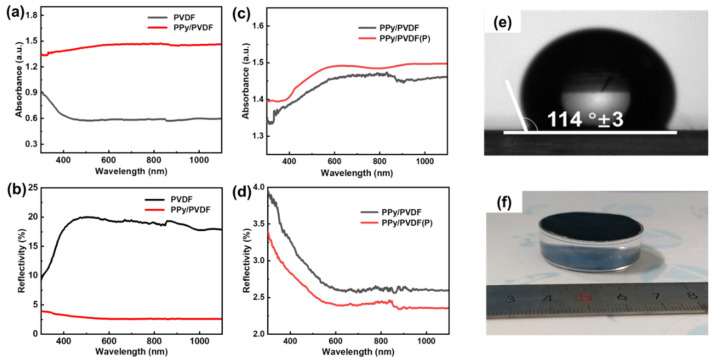
(**a**–**d**) Absorbance andreflectivity spectra of PVDF, PPy/PVDF and PPy/PVDF(P) membranes; (**e**) contact angle and (**f**) photo of self-floating PPy/PVDF(P) membrane.

Figure 5a,b shows the absorption and reflection spectra of PPy/PVDF and PVDF membranes. Compared to the PVDF membrane without PPy, the PPy/PVDF membrane has a great improvement in the absorption efficiency of incident light from 300 nm to 1100 nm. This is due to the fact that PPy is an excellent photothermal material with strong light-absorption ability. As shown in Figure 5c,d, the light-absorption efficiency of the PPy/PVDF(P) membrane with a micropyramid structure is higher than that of the PPy/PVDF membrane without a micropyramid structure. This is because the micro-pyramids of PPy/PVDF(P) can slow down the change of refractive index fromthe air to the membrane surface, thus effectively reducing the reflectivity, which conforms to the effective medium theory (EMT) [42,43]. Meanwhile, as PVDF is an intrinsically hydrophobic material, the contact angle of PPy/PVDF(P) reaches 114°, realizing its self-floating on the water surface, as shown in Figure 5e,f.

### 3.3. Optimization of PPy/PVDF Membrane

**Figure 6:**
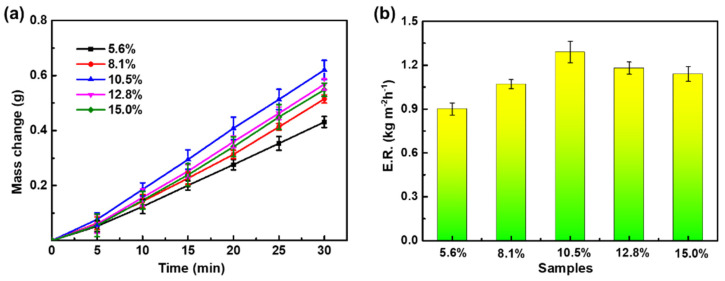
(**a**) Water evaporation mass and (**b**) evaporation rate of PPy/PVDF membranes with different mass fractions of PVDF.

The effect of the concentration of PVDF solution (DMF) on the evaporation rate of PPy/PVDF is shown in Figure 6. When other conditions remain unchanged, the mass fraction of PVDF is adjusted to 5.6%, 8.1%, 10.5%, 12.8%, and 15.0%, respectively. The corresponding evaporation mass of water is 0.4312 g, 0.5149 g, 0.6204 g, 0.5683 g, and 0.5481 g, respectively. The corresponding evaporation rate is 0.90 kg m^−2^ h^−1^, 1.07 kg m^−2^ h^−1^, 1.29 kg m^−2^ h^−1^, 1.18 kg m^−2^ h^−1^, and 1.14 kg m^−2^ h^−1^, respectively. With the increase of PVDF concentration, the evaporation rate of PPy/PVDF increases firstly and then decreases. This is due to the fact that the strength of PPy/PVDF membrane is not enough when the concentration of PVDF is low, and it will be deformed during the SVG process, which leads to the decrease of the evaporation rate. The pore size of PVDF membrane decreases with the increase of PVDF concentration. Therefore, when the concentration of PVDF is too high, the corresponding pores of smaller size will hinder the diffusion of water vapor, thus reducing the evaporation rate of water. In conclusion, PPy/PVDF has the best photothermal ability when the concentration of PVDF is 10.5%.

**Figure 7:**
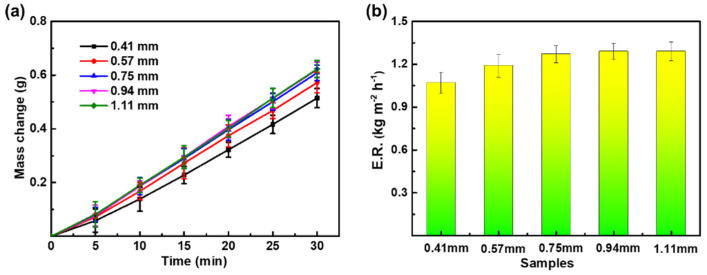
(**a**) Water evaporation mass and (**b**) evaporation rate of PPy/PVDF membranes with different thicknesses.

The effect of membrane thickness on the evaporation rate of PPy/PVDF is shown in Figure 7. When other conditions remain unchanged, the membrane thickness is adjusted to 0.41 mm, 0.57 mm, 0.75 mm, 0.94 mm, and 1.11 mm, respectively. The corresponding water evaporation mass is 0.5143 g, 0.5724 g, 0.6087 g, 0.6204 g, and 0.6228 g, respectively. The corresponding evaporation rate is 1.07 kg m^−2^ h^−1^, 1.19 kg m^−2^ h^−1^, 1.27 kg m^−2^ h^−1^, 1.29 kg m^−2^ h^−1^, and 1.29 kg m^−2^ h^−1^, respectively.

With the increase of membrane thickness, the evaporation rate of PPy/PVDF increases firstly and then remains constant. This is because the thin membrane facilitates the high transmittance of incident light, leading to the low light-absorption, which has a negative impact on the evaporation rate of PPy/PVDF. When the membrane thickness reaches 0.94 mm, the evaporation rate reached the maximum, and then remained almost constant with the increase of membrane thickness. In conclusion, when the membrane thickness is 0.94 mm, PPy/PVDF has the best photothermal ability.

**Figure 8:**
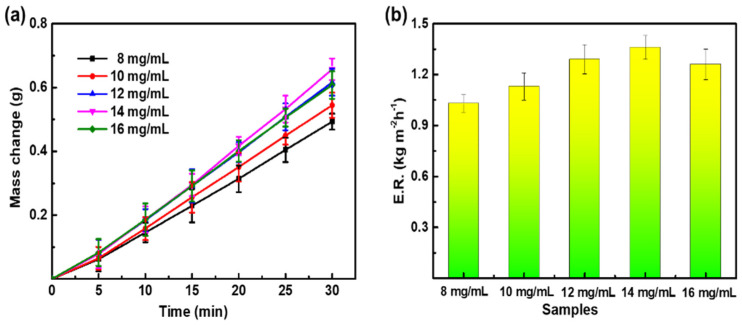
(**a**) Water evaporation mass and (**b**) evaporation rate of PPy/PVDF membranes with different PPy content.

The effect of PPy content on the evaporation rate of PPy/PVDF is shown in Figure 8. When other conditions remain unchanged, the content of PPy is adjusted to 8 mg/mL, 10 mg/mL, 12 mg/mL, 14 mg/mL, and 16 mg/mL. The corresponding water evaporation mass is 0.4931 g, 0.5446 g, 0.6165 g, 0.6573 g, and 0.6079 g, respectively. The corresponding evaporation rate is 1.03 kg m^−2^ h^−1^, 1.13 kg m^−2^ h^−1^, 1.29 kg m^−2^ h^−1^, 1.36 kg m^−2^ h^−1^, and 1.26 kg m^−2^ h^−1^, respectively. With the increase of PPy content, the evaporation rate of PPy/PVDF increases firstly and then decreases. This is due to that PPy is an excellent photothermal material, and the surface temperature of PPy/PVDF will increase with the increase of PPy content, thus speeding up the evaporation rate. However, the diffusion channel of water vapor is blocked when PPy content is too high, leading to the increase of humidity at the interface between PPy/PVDF membrane and water, which is not conducive to the subsequent evaporation of water. In conclusion, PPy/PVDF has the best photothermal ability when the content of PPy is 14 mg/mL.

### 3.4. Photothermal Property of PPy/PVDF(P)Membrane

**Figure 9:**
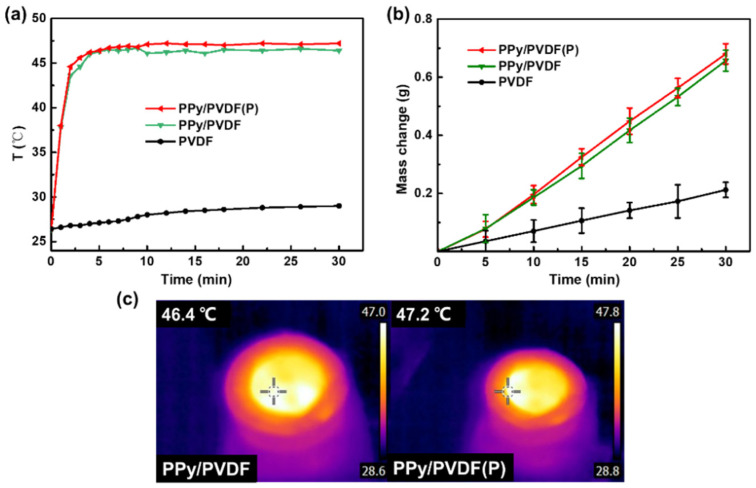
(**a**) Surface temperature and (**b**) water evaporation mass of PVDF, PPy/PVDF and PPy/PVDF(P) membranes; (**c**) infrared images of PPy/PVDF and PPy/PVDF(P) membranes.

The micropyramids on the surface of PPy/PVDF(P) membrane can further improve the photothermal ability. The equilibrium temperature of PPy/PVDF(P) membrane in the SVG process is higher than that of planar PPy/PVDF membrane, as shown in Figure 9a,c. This is because the micropyramids have the ability of antireflection, which can effectively improve the light absorption of PPy/PVDF(P) membrane, and thus enhance its solar energy efficiency. The high surface temperature of photothermal membrane facilitates the evaporation of water. Therefore, the evaporation rate of PPy/PVDF(P) membrane reaches 1.42 kg m^−2^ h^−1^, which is higher than that of the optimal planar PPy/PVDF membrane (1.36 kg m^−2^ h^−1^), as shown in Figure 9b.

**Figure 10:**
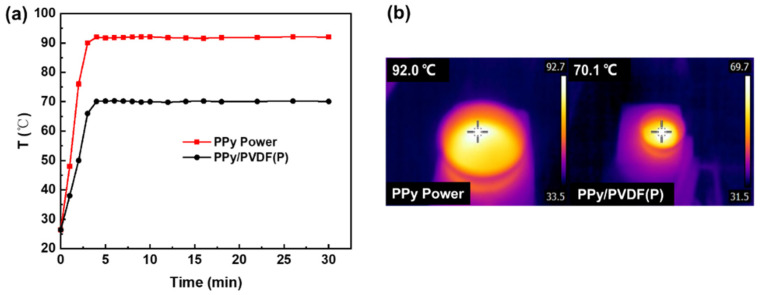
(**a**) Surface temperature and (**b**) infrared images of PPy powder and PPy/PVDF(P) membrane in dry condition.

Figure 10 shows the temperature change curve of PPy powder and PPy/PVDF(P) membrane without water evaporation under 1 sun irradiation. It can be seen that the surface temperature of PPy powder rapidly rises up to 92.0°C, which proves that PPy is a highly efficient photothermal material. Meanwhile, the surface temperature of PPy/PVDF(P) membrane without water evaporation also rapidly rises up to 70.1 °C, which is much higher than that of PPy/PVDF(P) membrane with water evaporation. The above phenomenon indicates that the heat generated by PPy/PVDF(P) during water evaporation can be taken away by the water at the solid–fluid interface, which is used for rapid water evaporation.

The photothermal conversion efficiency is a parameter to evaluate the utilization efficiency of incident light energy. The following formula is used to calculate [17,44,45,46]:

*H* = *mL_v_*/*P_in_* (1)

where *m* is the evaporation rate of water (kg m^−2^ h^−1^), *L_v_* (kJ kg^−1^) is the evaporationenthalpy of water, and *P_in_* is the optical input power (kW m^−2^). For PPy/PVDF(P) membrane, the enthalpy of water evaporation is 2248 kJ kg^−^^1^when the surface temperature reaches the highest (46.4 °C). Through calculation, the photothermal conversion efficiency of PPy/PVDF membrane is 84.9%. By using the above calculation method, the photothermal conversion efficiency of PPy/PVDF(P) membrane with micropyramids is 88.7%.

### 3.5. Salt Tolerance and Stability of PPy/PVDF Membrane

**Figure 11:**
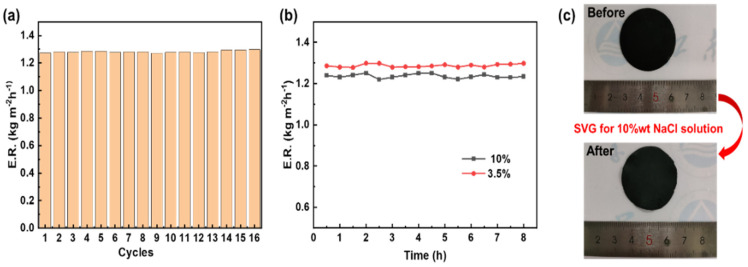
(**a**) SVG cycle experiment of PPy/PVDF(P) in 3.5%wt NaCl; (**b**) salt-tolerance test of PPy/PVDF(P) in 3.5%wt and 10%wt NaCl; (**c**) photos of PPy/PVDF(P) before and after 8 h SVG of 10%wt NaCl.

In order to prove the salt resistance and stability of PPy/PVDF(P), 3.5%wt NaCl solution (close to seawater) and 10%wt NaCl solution (higher concentration than seawater) were selected for the SVG cycle experiment. As can be seen from Figure 11a, the PPy/PVDF(P) membrane has good stability in the SVG process of 3.5%wt NaCl solution, during which the evaporation rate is basically stable at 1.28 kg m^−2^h^−1^ after 15 cycles of experiments. Figure 11b shows the evaporation rate change of 3.5%wt NaCl solution and 10%wt NaCl solution for PPy/PVDF(P) in the long-term SVG process. The higher the concentration of NaCl solution, the lower the saturated vapor pressure, resulting in the decrease of water evaporation rate. Therefore, the evaporation rate of 10%wt NaCl solution is lower than that of 3.5%wt NaCl solution, but it is basically maintained at 1.23 kg m^−2^h^−1^. Due to the hydrophobicity of PVDF, NaCl solution cannot enter into PPy/PVDF(P) membrane. Therefore, no salt is precipitated on the surface of PPy/PVDF(P) after 8hSVG of 10%wt NaCl solution (as shown in Figure 11c), showing excellent salt tolerance.

## 4. Conclusions

This photothermal membrane showed good self-floating ability in the process of water evaporation. In order to further improve the photothermal conversion efficiency, a micropyramid structure with antireflective ability was formed on the surface of themembrane by template method. The micropyramids can enhance the absorption efficiency of incident light. The water evaporation rate reached 1.42 kg m**^−^**^2^ h**^−^**^1^ under 1 sun irradiation, and the photothermal conversion efficiency was 88.7%.

The hydrophobic polyvinylidene fluoride ensures that NaCl cannot enter into membrane during the evaporation process of the brine, thus realizing the stability and salt resistance of polypyrrole/polyvinylidene fluoride in 3.5%wt and 10%wt NaCl solution.

A kind of hydrophobic PPy/PVDF(P) evaporator with self-floating ability, anti-reflective ability and long-term stability was fabricated by ultrasonic blending, phase separation and molding.

The micro-pyramid structure of the membrane surface can improve the absorption efficiency of incident light.

In the cyclic evaporation experiment of PPy/PVDF in brine, the salt cannot crystallize on the surface of PPy/PVDF(P) membrane, showing good salt tolerance and stability.

This structure and the preparation method provide a new idea to prepare long-term stable hydrophobic photothermal membrane.

## Data Availability

Not applicable.
